# Robert D. Acland – The Microsurgery Pioneer – A Personal Reflection

**DOI:** 10.1055/s-0045-1814458

**Published:** 2025-12-31

**Authors:** S. Raja Sabapathy

**Affiliations:** 1 Division of Plastic Surgery, Hand Surgery, Reconstructive Microsurgery and Burns, Ganga Hospital, Coimbatore, Tamil Nadu, India

Swami Vivekananda, the revered Indian monk and philosopher said,

“Take up one idea. Make that one idea your life – think of it, dream of it, and live on that idea. Let the brain, muscles, nerves, every part of your body, be full of that idea, and just leave every other idea alone. This is the way to success, that is how great spiritual giants are produced.”

Bob Acland lived up to the quote. He took up the idea of producing needles, sutures, and instruments to make anastomosis of vessels of 0.5 to 1 mm diameter possible. He was consumed by the idea, left off everything till he succeeded. He could be called the spiritual giant of microsurgery. By organizing a microsurgery training laboratory he made every one succeed, thereby making a difference in the lives of people worldwide.

Later in life he developed a second career in clinical anatomy. The passion, dedication, and devotion continued in this career and resulted in Dr. Acland's seven-volume video tapes in clinical anatomy, which he called as his “Sistine Chappel.” It is rare to find a comparison in the history of surgery of a person who has excelled in two different career paths.

**Fig. 1:**
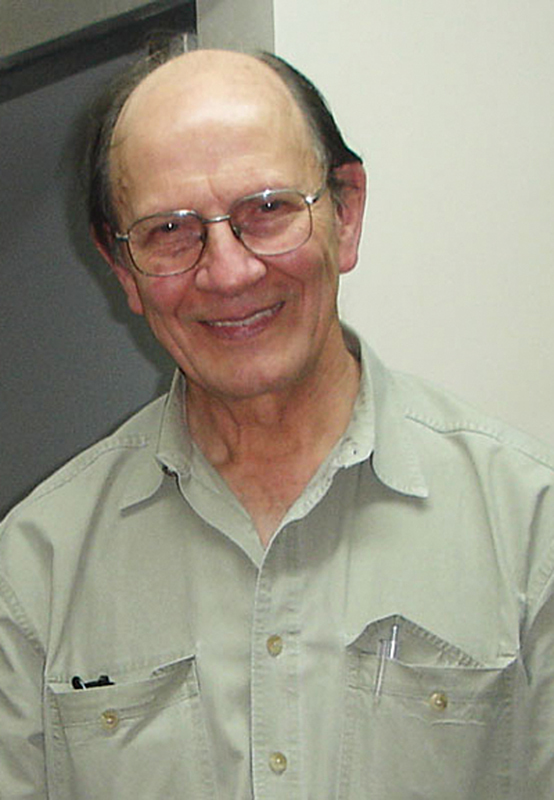
Robert D. Acland: 1941–2016, pioneer in microsurgery.

Robert Acland (Bob to his friends) was born on June 20, 1941, in Exeter, England, in an aristocratic family to Richard Acland the 15th Baronet of Columb John and Lady Anne Alford. Though the family belonged to the landed gentry, Richard Acland had far left ideas and believed in the common holding of land. He became a Member of Parliament of the Labor Party and later founded the Common Wealth Party, which believed in common ownership of land. True to what he propagated; he donated thousands of acres of the family estate at Killerton to the National Trust. Now without the family estates, Richard Acland told his sons that they would have to “make it on their own by being better, not by heredity.” Bob did become better by choosing a career in surgery ( Fig. 1 ).

**Fig. 2:**
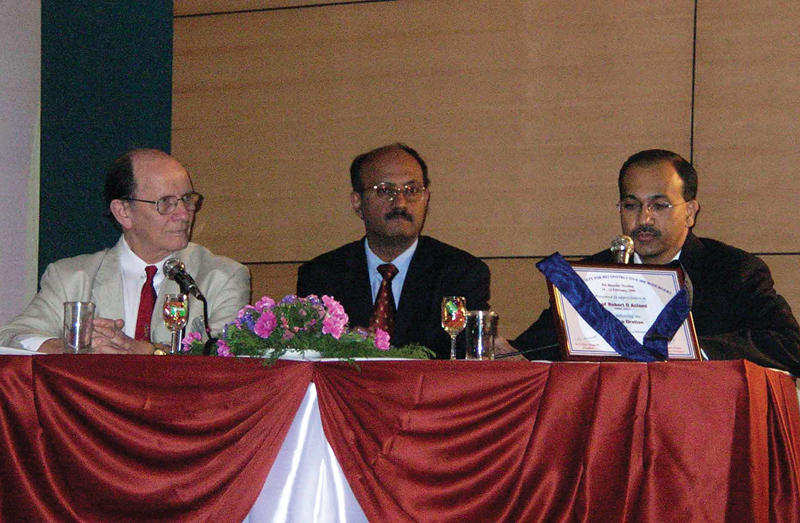
Dr. Acland at Indian Society for Reconstructive Microsurgery Congress, 2006, to deliver the Godina Oration with S. Raja Sabapathy, President, and Samir Kumta, Secretary, ISRM, Indian Society for Reconstructive Microsurgery.

Bob graduated from London Hospital in 1964 and had a year of internship in Tanzania. There he developed interest in surgery. He did the plastic surgery training in Canniesburn Hospital at Glasgow, which was the happening place of those times. In 1975, he moved to Louisville at the invitation of Harold Kleinert to set up a microsurgical training laboratory. Good and interesting accounts of his professional career are found in the obituaries that appeared in the journals at that time of his passing away. 1 2 3 I would like to devote the rest of the article to lessons learnt from the personal relationship with him and would like to express them with anecdotes. He came twice to India, first time in 2006 to deliver the Marco Godina Lecture at the Indian Society for Reconstructive Microsurgery meeting (Title: Anatomy - New Horizons in an Old Science) and in 2011 to deliver the Sushruta lecture (Title: Successes and Failures – a Pioneer's perspective) at the annual congress of the Association of Plastic Surgeons of India, both meetings being in Coimbatore when I held the presidencies of the organizations ( Fig. 2 ).

My first encounter with Acland was through his microsurgery training tapes. While working in Stoke Mandeville, United Kingdom, in 1988, before going to the microsurgery course at Northwick Park, my consultant Mr. Bailey told me, “Raja, Bob has presented a set of tapes. They are lying in the office among the books. Perhaps you could see them.” I saw them before going to the course. At the course, we were shown a demonstration of a microsurgery anastomosis which we watched in a television and were asked to go ahead. I was doing much better than many and decided that it was due to me having had the benefit of seeing the Acland tapes. I told this to Mr. Bailey and that Christmas while he gifted all registrars a bottle of Champagne, he presented me the “Red book” of Acland writing the words, “Learn to do it the right way.” Little did I foresee that one day I will have the privilege to join Dr. Acland to edit the third edition.

**Fig. 3:**
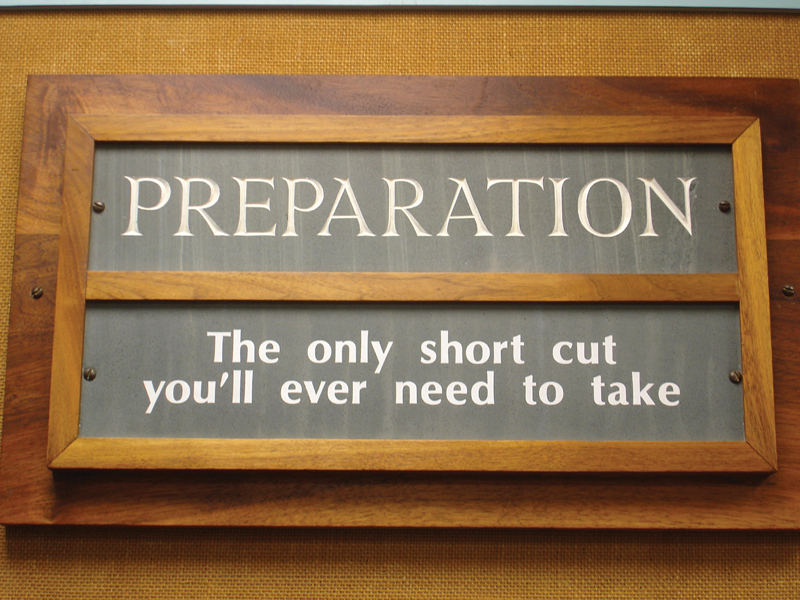
The sign found in the microsurgery laboratory at Louisville for all trainees to imbibe.

I was fortunate to obtain the Kleinert Fellowship at Louisville and there I first met Dr. Acland in 1989. Lot of stories about him preceded the course. I met him as I entered the foyer leading to the laboratory. At the entrance there was the word “Preparation” engraved in rock ( Fig. 3 ). While I stood looking at it, Dr. Acland joined and said that he wanted all the trainees to get ingrained in their brain that Preparation is the only shortcut to success. To lay emphasis he said he had it engraved in rock. That is Dr. Acland. He had a unique way of putting his points across.

He was benevolent. When we started the microcourse, I used to run it with the Acland tapes. The first tape got worn out. Dr. Luis Scheker from Louisville was coming to Coimbatore for the Apsicon 2003. Scheker wanted to know if I needed anything from Louisville. I asked for the first Acland tape. When Scheker asked Acland for a copy, Acland said, “I thought the tapes had outlived their value, I am surprised that someone is using them so far away. I don't have a tape to spare but I will do something.” In fact, he did. He converted the tapes from NTSC format and copied them on to a CD and sent it through Scheker. I was moved. He did not have to do that, but he did. Before sending he asked if we have any suggestions. He was egoless on that. The microsurgery tapes that were made in the early eighties are one of the longest surviving teaching tapes ever made in any specialty. Nearly half a century later the contents are still relevant. I think it was because so much thought and effort had gone into the making them a classic.

While working in Swindon in 1969 as a registrar, he was thrilled to see John Cobbett do a microvascular anastomosis. It struck Acland that things could be done with better instruments. Ever interested in making things, he set out to make better instruments, sutures, and needles for microsurgery. He got in touch with Harry Buncke almost two decades his senior by age who at that time was the pioneer in microsurgery in San Francisco. Both identified that there was a problem with the large size of the needle which left holes in the vessels of 1 mm in diameter. The progress seemed to stop there, but Acland was undeterred. Recollecting the situation in his Sushruta lecture Acland had this message for the young, “My colleagues told me that what I was trying to do was impossible. Don't be put off when someone tells you that what you are trying to do is impossible. The word impossible has a simple meaning. It actually means that I can't be bothered to find out the obstacles to success.” While delivering the Godina oration he showcased the fresh tissue cadaver laboratory at Louisville and then said that it is “a place to learn, a place to teach, a place to try new procedures and a place to do research.” Could it happen here? To anyone who says it couldn't, I suggest you take a small piece of paper and make a list of the obstacles to success.” For Acland no obstacle was intimidating.

**Fig. 4:**
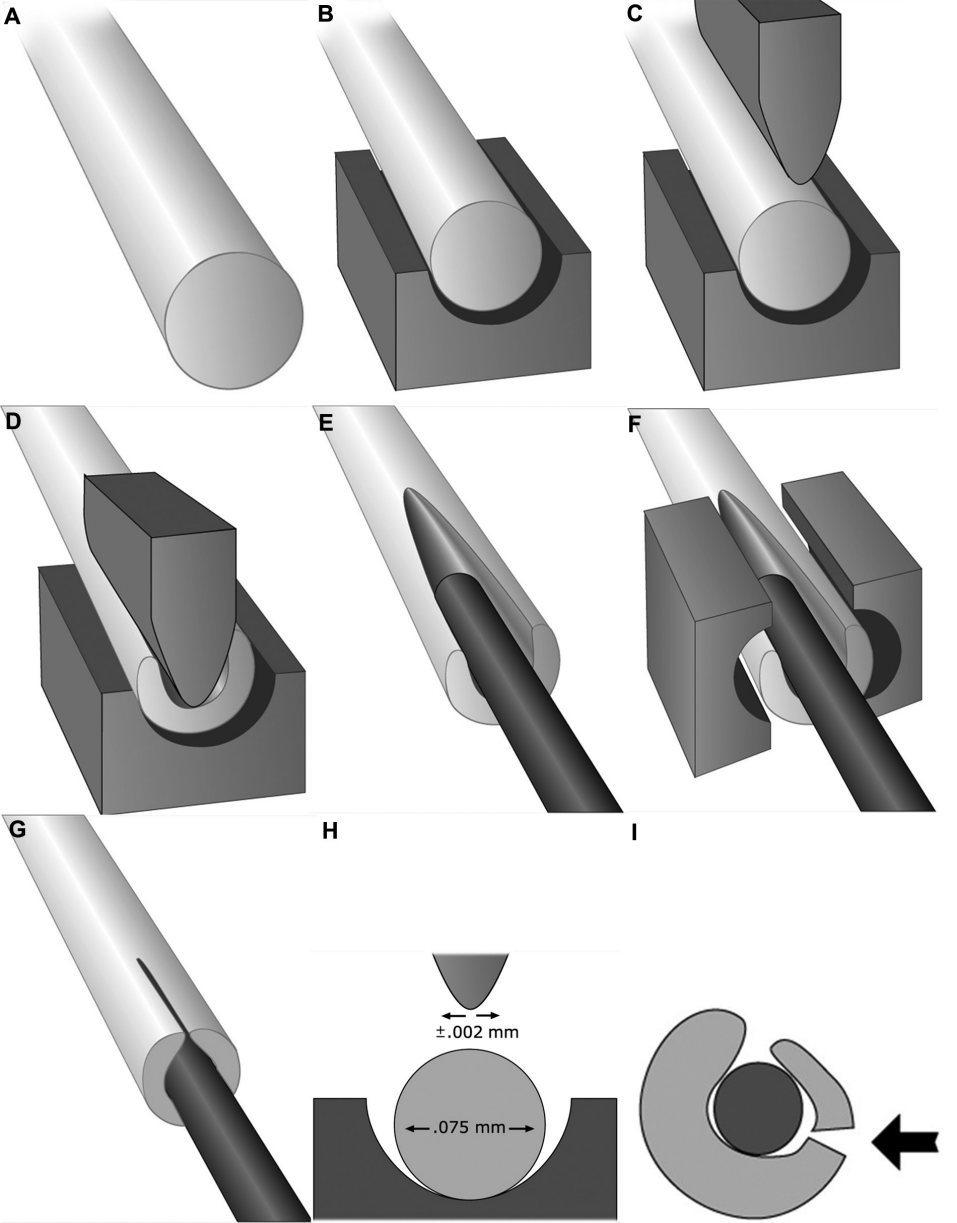
The breakthrough moment: ( **A** ) The needle, ( **B** ) placed in a bloc, ( **C** , **D** ) with an instrument to create a wedge, ( **E** ) for the placement of the suture and ( **F** ) another fine equipment to compress and ( **G** ) complete the process. ( **H** ) It required an accuracy of 0.002 mm to get a perfect suture. ( **I** ) Even minor deviation caused a breakage.

Acland wanted to make needles of 70 microns (0.07 mm) size. The smallest needle that existed at that time was more than twice that size. After searching to find a tool manufacturer who would help him he narrowed on a company founded by Werner Spingler and Eugene Tritt. in Switzerland. After a long correspondence Acland drove to Switzerland and landed at their doorstep, the founders exclaimed that they were expecting a senior British Professor and surprised to find a young man. Acland also exclaimed that he was expecting to land in a sophisticated Swiss factory, but instead a small set up. Soon Acland realised that Springler and Tritt were the people who could “make the tools and had the tools to make the tools.” The figures Acland used in his talk, explain the sophistication required to make it possible and swage the thread to the needle ( Fig. 4 ). The needle itself was made by taking the thinnest available stainless steel wire which was 100 microns. Acland used the technique of electropolishing to make the size to 80 microns. That was then passed through diamond burrs. In this he was helped by Ardelle Glaze. Acland said that we need to salute the contributions of those pioneers who put enormous effort to make this possible. He had these words of Thomas Brown for them,

“Who knows whether the best of men be known

or whether there be not

many more remarkable men forgotten

than any that stand remembered

in the known account of time.”

**Fig. 5:**
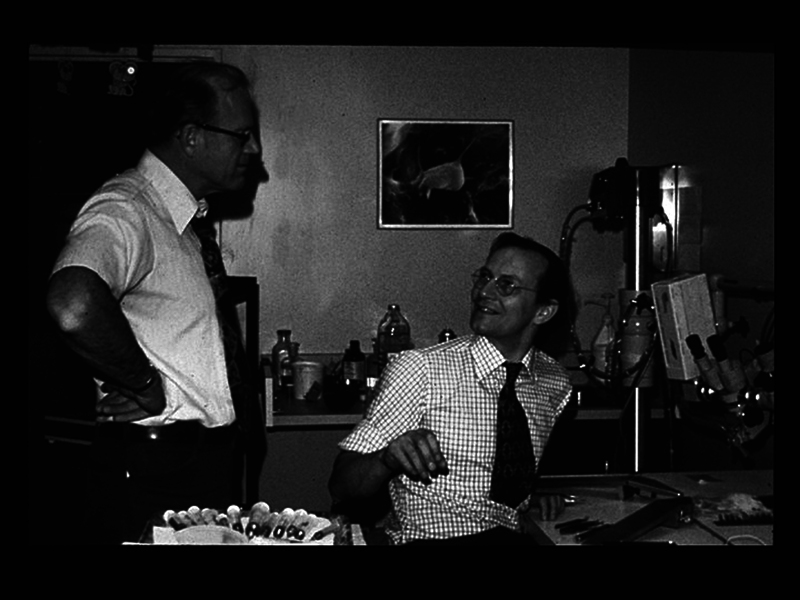
Acland with Harry Buncke (standing).

**Fig. 6:**
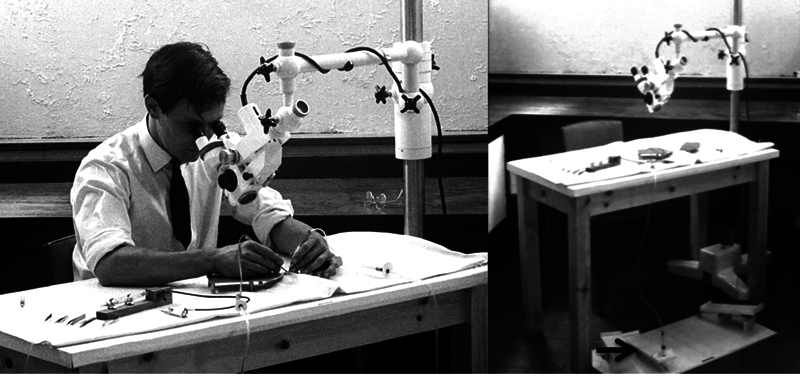
Acland experimented with a foot-controlled device to reduce coarse tremor for a year.

**Fig. 7:**
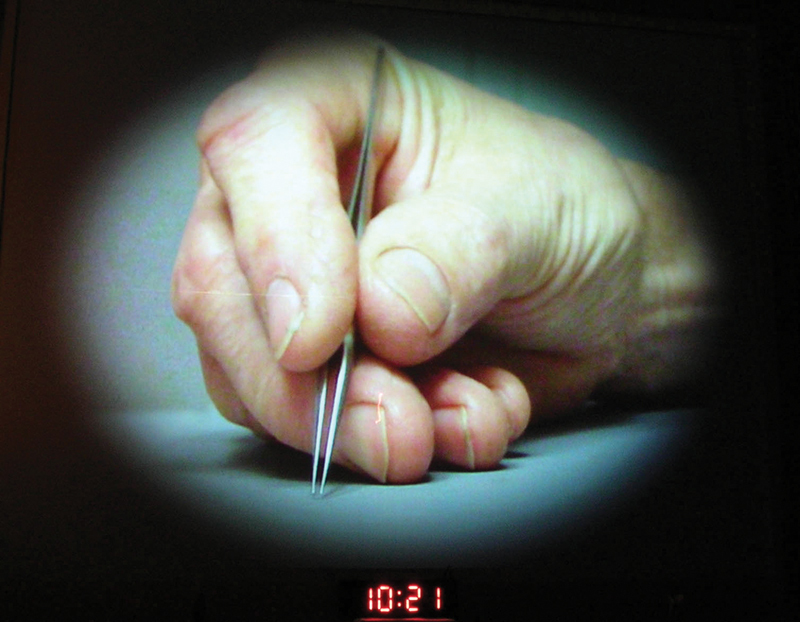
The defining moment in microsurgery as per Acland. Finding the hand and finger position to eliminate unwanted tremor. This led to designing of instruments.

Acland was also simultaneously working on designing instruments to do the fine work and controlling tremor. Sitting down, assuming a position of comfort, resting the ulnar border of the hand reduced coarse tremor, but fine tremor still persisted. Buncke and Acland ( Fig. 5 ) thought some remote-controlled equipment would be needed. For one year Acland experimented a foot-controlled technique to minimize tremor ( Fig. 6 ). Speaking of it he said, “It is good to have a lot of ideas but it helps to recognize quickly if it is a bad idea so that you can move on.” Finally, unintentionally he ended up finding the hand position—the hand and wrist well supported the little, ring, and middle stacked up one over another and the thumb, index, and middle touching one another manipulating the instrument. Acland said that this step was the defining moment of microsurgical progress ( Fig. 7 ). He added that “the answer to the problem when you find it will be very simple.” He also designed the clamp to hold 1 mm vessel without damaging the vessel wall.

Acland's relationship to India became stronger when he handed over the copyright of his “Red book”—Practice Manual for Microvascular Surgery which is considered by many as the bible for microsurgery trainees. Acland was concerned when the original publishers were not enthusiastic to reprint. During a casual conversation about this in the hospital canteen at Louisville, between Acland and Dr. Sunil Thirkanand, a staff surgeon at Louisville Hand Surgery, Sunil suggested that it could be published from India at an affordable price. Acland agreed and when Sunil wrote to me about the feasibility, I jumped at the opportunity. I was the secretary of the Indian Society for Surgery of the Hand (ISSH) at that time and thought that we would do it through ISSH. What started off as a casual conversation over a cup of coffee between Acland and Sunil turned out to be one of the biggest gifts to the Indian society for the surgery of the hand. Over 5,000 copies have been distributed and the proceeds have helped us to set up the Robert Acland International Travelling fellowship for young trainees.

**Fig. 8:**
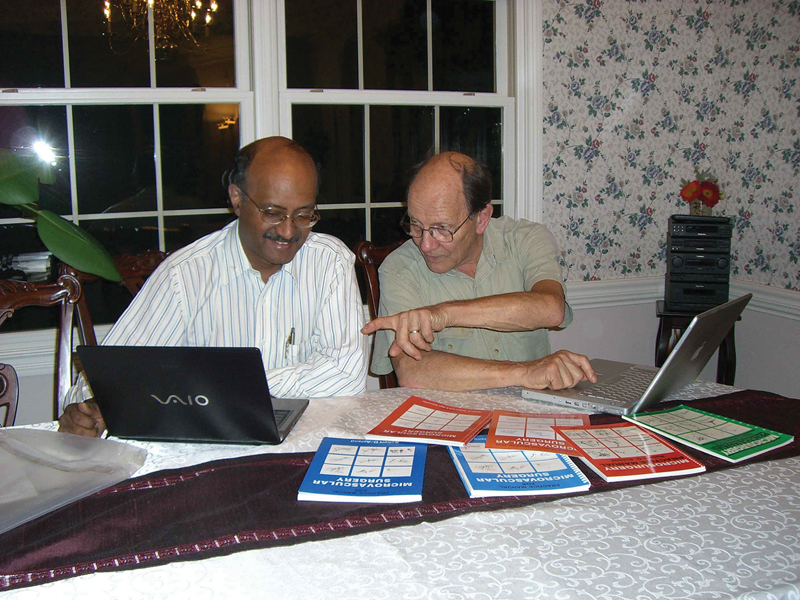
Editing the third edition of the book and experiencing obsession for perfection.

**Fig. 9:**
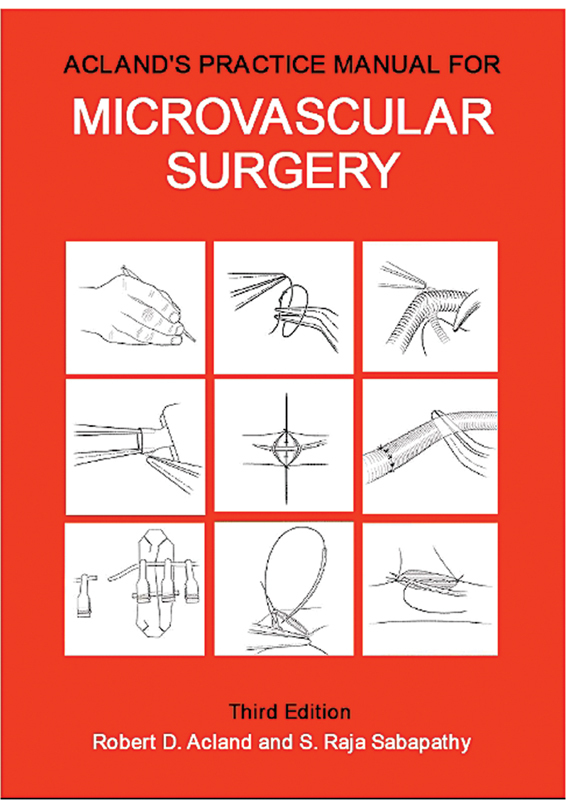
The Red Book.

Little did I realize how exacting it would be to work with Acland. Sunil did say that Dr. Acland would be demanding, but soon I started enjoying it. We reprinted starting from scratch since Dr. Acland wanted exact duplication of the material. Later, we decided that a chapter titled “Into Clinical Practice” could be added to make it clinically inclusive. Acland suggested that we meet when I next came to the United States. He drove down to Cleveland where I had gone for a meeting and agreed to stay at my cousin Dr. Maheshwar's house ( Fig. 8 ). We had a very long day from 8 in the morning to late night. For Acland every word mattered. We retired to bed late and when I met him the next morning, I casually asked him if he had a good night. To my surprise he answered, “No, partly because of you. When we were finishing last night, I asked you about a picture and you responded that it could be better. That bothered me and I worked on that picture. It took 3 hours. Have a look at this. How do you feel?” I was shocked. I never imagined that a casual remark would give him a sleepless night. That was Acland, the person obsessed toward perfection. Long hours did not matter to him. It had to be right. He really liked the final output ( Fig. 9 ). For the video atlas on clinical anatomy every minute of video took 12 hours to produce—5 hours for script writing, 5 hours for capturing videos, and 2 hours of postproduction editing. No wonder it took him almost 9 years to complete the project which he called his “Sistine Chapel.”

**Fig. 10:**
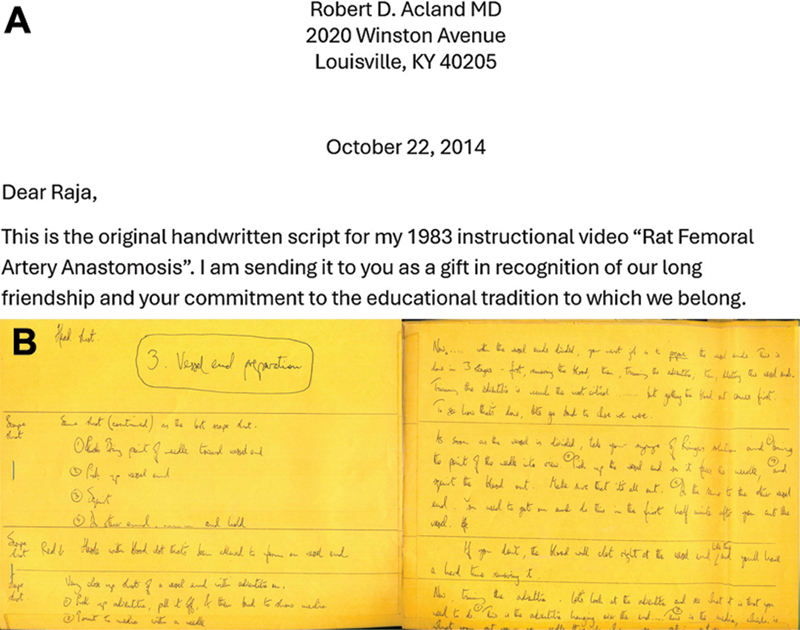
( **A** ) Offer letter of one of his precious possessions. ( **B** ) A model part of the script with the script on one side and corresponding video movement on the other side. A 10-minute video had ∼70 foolscap pages of written matter.

Acland set high standards for himself all the time even under difficult circumstances. He aimed to get that level of perfection even when working under constraints. I realized it when I received a letter from Bob Acland in October 2014. Bob decided to gift his “Precious Possessions” to his close associates. In that letter Bob had said that he wanted to gift the original hand written script of the 1983 instructional video of rat femoral artery anastomosis to me. That was the first instructional video he ever made. For making that video, on one side he had written the audio part of it and on the other side the actions that had to coincide. The script almost went to 75 pages. It had even the minor things like “smile,” “show it,” etc. ( Fig. 10 ). He wrote and corrected the spoken words and then created the video with care to make the actions coincide exactly with the words. Along with the letter there was a note stating “I cannot send it to you across the seas, you need to take it by hand as a hand baggage and not even as a check-in baggage.” He was so concerned that it should not be lost. So on my next visit for a meeting at San Francisco I went across America and collected it and it was my carryon baggage on my return trip to India. I consider it as one of the most valuable gifts I ever received.

Pioneers have a way of putting things and sharing their experience of the life's lessons that they have learnt. After his first visit in 2006, he wrote the following in our Visitors Book: “I am so happy to be here in your new United Nations of Microsurgical Progress and education. This feels the same in terms of energy and excitement as Louisville in 70s or Ljubljana in the 80s. My best wishes for your long continued success.” Later that night when I asked him what makes an institution fail and how careful one has to be? He was silent for a while. He then said, “After thinking on the rise and fall of institutions during my lifetime I would consider these as the chief causes.

1. Celebrating success
2. You will be training the world but fail to train people who will be with you.
3. Forgetting the core values which brought you up to this level in the first place.

He continued, “Raja please be careful, that once you succeed, people will keep asking for your story. You tell the story but let that not become your preoccupation. You must have your feet on the ground and continue to work in the same pace. Be careful that you continue to have star performers in the team, who share the same passion for work and education. Lastly, you must not forget the core. People may say that times have changed and justify change in values. You may choose different methods or use technology to achieve the core, but the core values should not change.” It was like hitting a hammer on my head. During the Sushruta oration at the APSICON 2011, he highly appreciated the technical refinements suggested by Dong Chul Lee (South Korea) to evert the vessel walls during anastomosis and wondered why he had not thought about it himself. Reflecting that he had this to say “….coming back to the theme of success and failure, when I reached a certain level of success, I failed to be self-critical, I failed to keep looking for ways to do even better. That is the potential risk that we all face when we achieve success.” These are timeless values spoken in simple words.

People remember Bob Acland as per the experience they had with him. Gus McGrouther, who joined Canniesburn had this to say of Acland. “The sheer force of his personality resulted in a multitude of apocryphal stories, often making it hard to disentangle truth from fiction.” But everyone will attest to the fact that he was full of passion and enthusiasm to create something better. Scott Levin, Past President of the World Society for Reconstructive Microsurgery, had this to say. “Bob Acland was fiercely dedicated to the profession and “attention to details” were his watchwords. Bob was a modern Renaissance man. He was as comfortable restoring a 60-year-old pickup truck that served as his “daily driver,” as he was building special platforms that he used to display his masterful anatomic dissections that he photographed himself. *Bob “did not suffer fools” and his passion for perfection in everything he did, often caused him to be misinterpreted and feared.* My passion for anatomy and microsurgery was born in Louisville and has been fuelled for 37 years by the legacy of Bob Acland. Principles and memories are forever, and it is true that we stand on the shoulders of giants.”

**Fig. 11:**
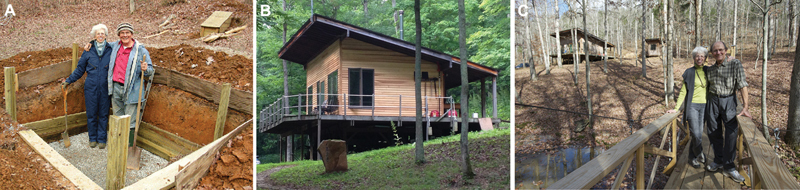
Acland was energetic and enthusiastic in construction, carpentry, and machine making. ( **A** – **C** ) The terrace house and the walkway he built himself with his wife Bette.

The other side of Bob is also very interesting. He was crafty with his hands and enjoyed construction to carpentry to automobile engineering. He spent the last days of his life constructing a one-room house above the “Mosquito Creek' in Indiana and created water, heating, and lighting systems by himself and also built a suspension bridge for access ( Fig. 11 ).

**Fig. 12:**
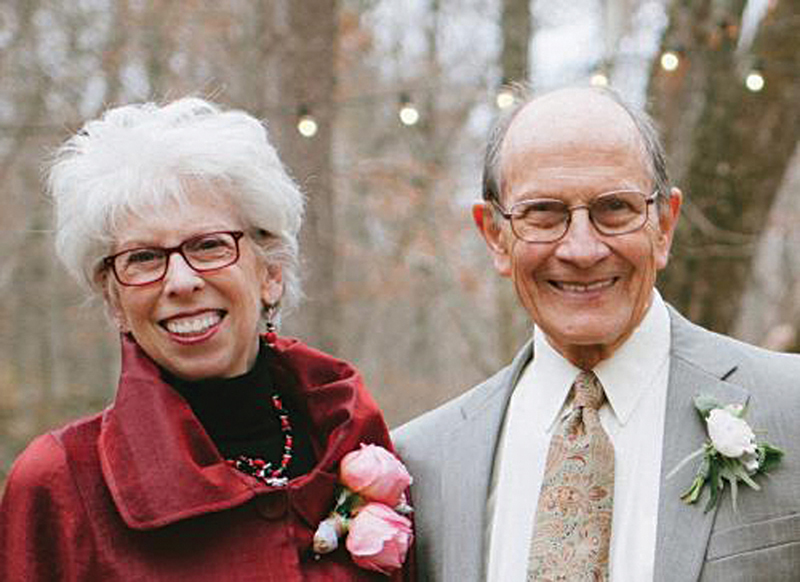
Bob with his wife Bette.

Acland loved India and after his visit in 2006 said that he had one regret. That he had not brought his wife Bette with him. Bette accompanied Bob when he came in 2011. He wanted to make the same tour of Kerala that he experienced. As luck would have it we organized the same car and driver and hosted them in the same hotels. Some hotels even made the same staff attend on him. When I wrote to his wife Bette Levy that I am writing a note on Bob for the icon of the issue of the *Indian Journal of Plastic Surgery* she sent this message ( Fig. 12 ). “As the wife of Robert Acland, I shared 25 years filled with love, playful humor, laughter, and a joy of gardening, movies, travel, good food, and good friends. We shared a sense of the absurd and were known for our annual New Year's Day costume party. While all love relationships are different, our love was palpable and frequently commented upon by those who knew us. We delighted in our Southern Indiana creek house, lovingly built by hand by Robert, and where we spent many contented and peaceful days and nights.”

I had the opportunity of studying the lives of pioneers in making the book, “Crafting the Legacy,” where we compiled the life stories of pioneers in hand surgery in the Asia Pacific region. 4 Common to them all is a deep desire to help their patients, All had laser sharp focus on the job, infectious enthusiasm, relentless reflection on all that they did, and left behind an enduring legacy. 5 6 I would say that Acland comes up high on my list of heroes. Behind every pioneer is a story that will stir the hearts of others. These need to be told to enthuse the younger generation, so that they would do the same of their patients and in turn, we will all have a better world to live in.
